# A qualitative study exploring participants’ experiences of the SCOPE2 trial: chemoradiotherapy dose escalation in oesophageal cancer

**DOI:** 10.1186/s13063-025-08768-z

**Published:** 2025-02-26

**Authors:** Daniella Holland-Hart, Mirella Longo, Sarah Bridges, Lisette Nixon, Maria Hawkins, Tom Crosby, Annmarie Nelson

**Affiliations:** 1 Division of Population Medicine, Cardiff University, Neuadd Meirionnydd, Heath Park, Cardiff, Wales CF14 4YS UK; 2 Centre for Trials Research, Cardiff University, Neuadd Meirionnydd, Heath Park, Cardiff, Wales CF14 4YS UK; 3 University College London, Medical Physics and Biomedical Engineering, London, WC1E 6BT UK; 4 Velindre NHS Trust, Velindre Road, Cardiff, Wales CF14 2TL UK

## Abstract

**Introduction:**

This qualitative study explored patients’ experiences and perceptions of the SCOPE2 trial. The trial studied radiotherapy dose escalation in patients with inoperable oesophageal cancer treated with definitive chemo-radiation. SCOPE2 embedded a phase II trial for patients with a poor early response using positron emission tomography (PET) scans.

**Methods:**

This longitudinal interview study took place between 2017 and 2021. Patients eligible for chemoradiotherapy were recruited from five clinical sites in the UK. Participants were invited to participate in three semi-structured interviews across four different time points: baseline (before treatment) and at 2–3 months, 3–6 months or 6 months + after baseline. This paper focuses on recruitment to the trial, practical management, the impact of COVID-19 and reflections of being on the trial. Real-time reporting to the trial team was used to inform potential improvements to trial conduct and recruitment. The interviews were thematically analysed.

**Results:**

Ten participants were interviewed in 16 longitudinal interviews. There were five female and five male interview participants; three participants were accompanied by companions during their interviews. Recruitment to the trial and qualitative study was challenging. Motivations for joining the trial included altruism, potentially receiving better care and monitoring and the opportunity to improve their quality of life. Participants required adequate time to consider information and regular updates regarding trial and treatment process. Participants felt that their trial experience was minimally impacted by COVID-19, although some delays to treatment were reported.

**Conclusion:**

Increased opportunities for patients to discuss and receive appropriate and timely information from trial staff and third sector partners could enhance patients’ understanding of future trials, treatments and procedures. Slow recruitment to the trial and qualitative study was further impeded by the COVID-19 pandemic and future trials would benefit from a more fully integrated approach to qualitative recruitment.

**Clinical trial registration:**

ClinicalTrials.gov: NCT02741856 registered on 12 April 2016; ISRCTN: 9,712,546 registered on 26 October 2016.

**Supplementary Information:**

The online version contains supplementary material available at 10.1186/s13063-025-08768-z.

## Background

Most patients diagnosed with oesophageal cancer (OC) who are eligible for curative treatment but are ineligible for surgery are typically offered chemoradiotherapy treatment (CRT) [1]. Previous trials have found that standard chemoradiotherapy can improve longer-term survival, quality of life and reduce toxicity for patients with curative OC [2–4].

SCOPE2 (trial number: NT02741856; ISRCTN: 97,125,464) is a randomised phase 2/3 trial which examines radiotherapy dose escalation in patients with OC treated with definitive chemo-radiation, and is ongoing at the time of writing [5]. Patients usually receive 50 Gy of radiotherapy alongside chemotherapy as standard practice, but in SCOPE2 half of the patients are randomised to receive a higher dose of 60 Gy alongside concurrent chemotherapy. SCOPE2 embedded a phase II trial exploring a second PET scan taking place 14 days after the start of chemotherapy. Those patients who had not responded sufficiently to their initial 2 weeks of chemotherapy were included in a randomisation to either continue with their initial chemotherapy regimen, or switch to a different chemotherapy regimen for the remainder of their treatment. The PET sub-study was closed in August 2021. SCOPE2 follows the SCOPE-1 trial (2013) [4] which assessed a range of areas including quality of life and feasibility of recruitment. However, the SCOPE-1 trial did not explore patients’ self-reported experiences of the trial and treatments.

Qualitative studies integrated in clinical trials have highlighted patients’ experiences important to trial conduct and feasibility which are not captured through quantitative measures. These include patient preferences in a non-inferiority trial [6], issues of clinical equipoise in a feasibility trial that failed to recruit, strategies for recruitment in a primary care trial, and participants’ understanding of complex trial processes in a stratified trial of personalised therapies [7, 8]. In-depth insights into the experiences and challenges of patients with OC relating to trial recruitment, patients’ experiences and outcomes have also been highlighted in previous qualitative studies [9, 10]. These included the extra time, energy, side-effects and financial cost burdens of trial participation. This qualitative study therefore sought to explore the first-hand experiences of participants which were not captured through clinical and quantitative data.

## Methodology

This multi-centre, qualitative interview study explored the real-time, longitudinal experiences and perceptions of people with curative OC within the UK-based SCOPE2 RCT before, during and after chemoradiotherapy treatment. This includes both patients who were included in the PET sub-study and those who were not.

This paper discusses participants’ experiences and perceptions including trial recruitment, conduct and practical management. Patients’ experiences of treatments and their impact on participants’ health and wellbeing are reported in Holland-Hart et al. [11].

### Aims

The qualitative study aimed to explore the real-time experiences and perceptions of SCOPE2 participants to inform the trial and future trial practices.

#### Objectives

- To assess patient experience and perceptions of recruitment and practicalities of being on the trial
- To understand the impact of COVID-19 on patients’ experiences of the trial
- To understand patients’ reasons for declining the trial
- To report real-time patient experiences to the main trial

### Recruitment

The main trial began in 2016, it was paused due to the COVID-19 pandemic between March and August 2020, recruitment was completed in December 2023 and closed in January 2024. Recruitment to the qualitative study was open in 2017, paused between March and October 2020 due to the COVID-19 pandemic and then fully closed in December 2021. Qualitative data was collected from patients over 17 years old, eligible for curative definitive chemoradiotherapy, from five of the trial sites open to the qualitative study across the UK. Each clinical site open to the qualitative element was provided with initial and refresher training by the qualitative team regarding the processes and purpose of the qualitative study. Training was provided either face to face or online, usually lasting around 20 min each session. Training materials were also available in digital forms for recruiting staff to revisit at any point.

Participants were informed of the optional qualitative interview study at the point of consent into the main trial or up to 24 months after and were provided with a qualitative study information sheet (PIS) and consent form (CF). If patients wished to participate in the qualitative interviews, they could either provide consent during a trial appointment after which, patient’s contact details via secure email were sent to the qualitative researchers. Alternatively, patients were presented with a tear-off slip and a stamped addressed envelope to provide their contact details to the qualitative team directly. The qualitative researchers contacted patients to arrange an interview, and then during the interview patients provided signed consent. Companions who accompanied participants during interviews were also required to complete a consent form. Participants were invited to participate in up to three interviews. Patients who declined the main trial were also invited to participate in an interview with the qualitative team and provided with a PIS and CF.

The original sample size of 30–40 qualitative study participants was based on the homogeneity of the population, and the aim of understanding experiences equally distributed across the different trial arms. However, regulatory approvals for opening the qualitative element of the trial and site level access were granted much later than to the main trial; these, along with wider barriers to recruitment (e.g. COVID-19 pandemic), resulted in delays and lower numbers of participants than sought and opportunistic rather than purposive recruitment.

Due to slow recruitment to the qualitative study between 2017 and 2018, members of the recruiting teams at participating sites were asked in late 2018 for informal feedback to describe the barriers to recruitment. Reasons provided for this slow recruitment to the qualitative element of the trial included health issues of patients and lack of available staff for recruitment. Recruitment to the main trial was also hindered by concerns about increased dosage of radiotherapy; patients preferring standard care due to extra burdens of treatment and information overload; and adhering to timelines for PET scans, which consequently impacted qualitative study recruitment. Following this feedback, approval was granted in 2020 for the qualitative research team to interview patients up to 24 months after recruitment to allow extra time for patient participation. Amendments were also made to the PIS to accommodate remote interviewing, as face to face interviews were not viable due to the COVID-19 pandemic.

### Data collection

Semi-structured interviews were carried out by the first and second authors, who are social researchers with experience of interviewing participants on sensitive topics including in cancer trials. These researchers worked outside of the main trial team, therefore were able to collect and analyse the data from a critical perspective, with minimal preconceptions about what participants should have expected. Before social restrictions were imposed in March 2020, interviews were carried out face to face, after which they were conducted by telephone. Patients were only contacted for initial or follow-up interviews, after the qualitative researchers checked with relevant research nurses at each site as to whether the patients were still able and willing to participate. Due to the short timeframe between recruitment and treatment, and the complex consent processes, it was not always possible to interview participants at baseline; in these circumstances, participants were asked to recollect their trial recruitment in later interviews. Participants’ demographic information was collected and reported descriptively but was not used as sampling criteria due to the low numbers of participants. Participants were not provided with payments for taking part.

Semi-structured interview guides were used to ensure the main topics were covered across the interviews but also allowed for discussions to be guided by the participants. After recruitment restarted in October 2020, a revised version of the interview schedule included additional questions aimed at exploring the impact of the COVID-19 pandemic participants’ trial experiences (interview schedules Supplemental files 1, 2). Interview topics included recruitment to the trial, practicalities of being on the trial, the impact of COVID-19 on their experience of the trial and reflections on the trial. All interviews were audio recorded using an encrypted digital recorder and transcribed verbatim.

### Data analysis

Participants’ experiences of specific time points pre- and post-treatment were captured through the longitudinal interviews [12]. These qualitative data sets were thematically analysed using an adapted form of Braun and Clarke reflexive thematic analysis [13] and development of a codebook [14]. This modified approach was necessary to inductively analyse the data, while seeking to address specific trial focused questions. The analysis initially involved interpreting the interview data, generating open codes and identifying patterns. All data were coded by the lead researcher and 20% was coded separately by the second author; then, codes were agreed on. Themes were generated by combining relevant codes and identifying overarching concepts. A codebook was then established to organise the themes and subthemes, using NVivo™ software program for data management. Key themes were agreed on and presented in the results, relevant to the research questions.

Key findings regarding participants’ real-time experiences of trial processes and treatment protocols were reported to trial management meetings to influence necessary changes to trial procedures and practices with the aim of improving recruitment and patients’ experience. Further information regarding this qualitative research study is reported in the COREQ checklist (Supplemental file 4).

### Public and patient involvement (PPI)

Two research partners (patient representatives) oversaw the main trial by providing assessments of trial documentation, assisted with Scientific Milestone Reports, and contributed to TMG (Trial Management Group) meetings. This included reviewing the qualitative study’s interview guide, patient information sheets and main findings.

## Results

### Participants

Sixteen longitudinal interviews were conducted with ten participants. Participants were interviewed up to three times across four different time points: baseline (after consenting to the trial and before treatment), 2–3 months, 3–6 months or 6 months + after baseline (all interviews took place within 7 months after baseline). Five females and five males participated, this compared to 62.2% males in the main trial, which is more aligned with distribution across the population (69% males) [15]. Three participants were joined by companions. Participants were between 57 and 82 years; the mean age of the participants was 70 years. This compares to only 24.6% of SCOPE2 trial participants 75 > , which is lower than UK 41% average [15]. Demographic information was collected by the main trial, limited to age and gender. Five participants received one PET scan and five received two PET scans. A participant was known to have died after three interviews. Before the qualitative study was paused in March 2020 pre-COVID-19, seven interviews took place in person with four participants. Nine interviews were conducted via telephone with six participants post-October 2020 after the qualitative study was given permission to reopen. Information regarding participants’ study arms, interviews and demographics is also illustrated in Table 1. There were no patients recruited to the qualitative study who declined the main trial.

**Table 1:** Participant study arms, interviews and demographics

Participant	Study arm	Gender	Age range	Baseline	Between 2 and 3 months after baseline	3–6 months after baseline	6 months + after baseline	Companion attended	PET scan at day 14	Death
Participant 1	2	M	80 +		X	X		X		
Participant 2	2	F	70–79			X			x	
Participant 3	1	F	70–79		X			X	x	
Participant 4	1	M	50–59	X	X		X			x
Participant 5	1	F	70–79			X				
Participant 6	4	F	60–69	X		X	X			
Participant 7	2	M	60–69			X	X		x	
Participant 8	3	F	50–59			X			x	
Participant 9	2	M	70–79				X		x	
Participant 10	4	M	80 +	X				X		

Interviews were conducted with patients from across the trial’s four treatment arms: standard dose dCRT prescribed carboplatin/paclitaxel (arm 1); standard dose dCRT prescribed cisplatin/capecitabine (arm 2); escalated dose dCRT prescribed carboplatin/paclitaxel (arm 3); and escalated dose dCRT prescribed cisplatin/capecitabine (arm 4) (Table 1).

Key findings from the interviews relate to the following themes: recruitment to the trial, understanding trial information, practicalities of being on the trial, the impact of COVID-19 pandemic and reflections on the trial. These findings are outlined below supported by relevant quotations; additional quotations are also available in supplemental 3 file. The themes and subthemes in this paper are presented in Table 2.

**Table 2:** Themes and subthemes

Themes	Subthemes
**Recruitment to the trial**	Motivations to join the trial
	Decision to join the trial
	Understanding the trial information
**Experiences of being on the trial**	Practicalities of the trial
**Impact of COVID-19 pandemic**	
**Reflections on the trial**	

### Recruitment to the trial

#### Motivations to join the trial

Participants often described altruistic reasons for participating in the trial; several were motivated by the possibility of contributing to research, which could improve treatments and services for future generations. Gratitude and a sense of moral duty towards the NHS were also expressed as key motivations for joining the trial.

I really do think trials are an absolute necessity. Participant 6 (Baseline)

I was pleased to be asked. Delighted to help national health, I had so much wonderful treatments… if I can give some help back then I am absolutely very pleased to do that. Participant 3 (2–3 months)

Some participants felt that potentially receiving better care and monitoring throughout the trial also encouraged their participation.

I think we were given indication that there might be an element of better, fuller care. Participant 1 (2–3 months)

Joining the trial at times offered hope to participants who had limited treatment options available. Despite some misgivings about chemotherapy, one patient explained that the trial offered a chance for them to regain their basic quality of life.

I don’t like the idea of destroying all the cells in my body and starting again, no, did I not, but it needs to be done, otherwise I’ll never eat again. Participant 8 (6 months)

#### Decision to join the trial

The provision of sufficient time to consider the trial and discuss and confirm its details with staff was considered important, as patients felt that it could be difficult to absorb all of the information at once, particularly during the challenging time around diagnosis. Also, information provided by healthcare professionals about being able to stop participating, reassured patients about joining the trial.

The day I made the decision, I don’t think I was taking anything in, but I knew I was making the right decision. I didn’t even look at the paperwork for about another week because there were so many things I was trying to get into my head. So, when [consultant] did ring me, I had a long discussion with him about it, and then I went back to the paperwork. So, I think … it’s a good idea not to frontload people with too much information. Participant 6 (Baseline)

These challenges were fed back to the TMG, resulting in the production of an introductory PIS flier provided at an earlier time point in the recruitment process, which supported the incremental presentation of information rather than altogether.

#### Understanding the trial information

Information provision, particularly at the beginning of the trial, was perceived as generally useful and patients reported satisfaction with their understanding of the trial and treatment options. Participants felt reassured by the opportunity to discuss their ongoing concerns with their hospital trial contacts.

The doctor explained it fully and they had a lead nurse that continued, I had forms to fill, things like that and they explained fully what was going to happen. Participant 9 (6 months)

More timely and thorough information would have been helpful to some participants, who were at times unclear about what would happen before, during and after their treatments. This particularly related to how much time the treatment process would take, as they were not always prepared for the length of time, or amount of organisation and resources that were required, especially in relation to radiotherapy. These concerns were more apparent in later interviews, as participants had time to reflect on any information deficits.

I am completing the radio this week and the chemo care is on next week… and that’s the end … Precisely what happens then, the natural order is and how long they may take and what dates they might be for check-ups and endoscopies and so on. We are little big vague on that. Patient 1 (2–3 months)

As a result of qualitative feedback, the TMG aimed to improve updates throughout the trial via incremental information provision during participant appointments, as well as regular newsletters updating participants on trial progression.

### Experiences of being on the trial

Being informed about what would happen during procedures such as PET and CT scans was considered important, as participants felt that full explanations helped allay their concerns. Lack of preparedness for the length of the procedures such as CT scans, as well as insufficient care regarding comfort of the patients during scans, was also reported.

There wasn’t any great care taken to allow for [my] hunch... It was very uncomfortable, and I still got trouble with this arm consequently… certainly I wasn’t told…[A] Little bit more could be said, about lengths of time and divisions of time. Patient 1 (2 months)

#### Practicalities of the trial

The pace at which the treatments proceeded once patients joined the trial was perceived as highly efficient. The high quality of care and efficiency of the testing service was also described.

Since I’ve been to Oncology… everything has just been zoom, zoom, zoom… The days I went for all the tests, I felt like a VIP, I was being whisked to the bloods, and then to ECG … there was no waiting for anything, everybody was expecting me … I was very impressed. Patient 6 (Baseline)

### Impact of the COVID-19 pandemic

Participants reflected on the varying impacts that the COVID-19 pandemic had on their trial experiences. Some felt that their trial experience had not been negatively impacted, whereas others described how the pandemic had delayed their diagnosis, treatment start dates or access to treatments.

I don’t think it affected the … the trial … my problem was the initial diagnosis … there was a lot of delays there. Participant 6 (Baseline)

I had Covid, and it was difficult at times in hospital because of the restrictions you see, and you had to be so careful, and I think it caused a bit of delay in the radiation because you had to wait for a while, but other than that it was inconvenient, but it did not cause any problems with the treatment. Participant 9 (6 months)

### Reflections on the trial

A sense of gratitude was expressed by certain participants when reflecting on their opportunity to participate in the trial. The extra monitoring, personalised support and quality of care that they had received from the NHS and third sector professionals was perceived as a particularly positive aspect of participating.

I have felt that the benefit of the trial is that little bit of extra monitoring, that little bit of extra care. Participant 4 (6 months)

The medical staff have really been great, and ... I’ve got all the information … [all] I need to do is pick the phone up and I know I can speak to somebody with any questions … I have been in contact. Patient 7 (3 months)

Overall, these longitudinal interviews highlight that participants’ experiences of the trial were generally consistent over time.

## Discussion

This qualitative study reported patients’ reflections on recruitment, trial information and overall experience of the SCOPE2 trial. The real-time reporting of participants’ experiences to the trial team informed of the ongoing communication, physical and social needs of patients, described in Holland-Hart et al. [11]. This led to the adoption of new communication strategies for participant information provision throughout the trial, which will also inform future trials.

Slow recruitment to the trial was exacerbated by the COVID-19 pandemic, reflecting challenges in recruiting to cancer trials more generally during this time. Recruitment to cancer trials in the UK fell by nearly 60% during the first year of the pandemic [16]. Reported barriers to recruitment for the SCOPE2 trial also included delays in set up times due to site’s resources, lack of patient eligibility and time pressures due to the complex consent process [5].

Difficulties in recruitment to the qualitative study, particularly relating to those who declined the trial, differed to the main trial. Strategies to enhance recruitment to qualitative studies in trials include reinforcing the value and benefits of qualitative research and working with PPI to engage identify the needs of patients [17]. Nonetheless, changes to qualitative data collection methods during the pandemic from face-to-face to telephone interviews likely improved uptake. This enhanced interview efficiency in terms of costs, time spent on travel, and opportunities to reschedule, also conduct relating to privacy, reflecting prior studies [18, 19]. Interview responses were equally as rich and ensured less health risks to participants and researchers. This contrasts with the view that remote interviews are generally less rich, generating fewer field notes but still producing substantive coding [20]. Highlighting that data collection approaches, particularly with clinically vulnerable cohorts should be context dependent, and where possible flexible.

Despite altruism being a significant motivator for participation in this trial [9, 10], some individuals also perceived that they would experience some benefit and suffer no disadvantage [21–24]. This reflects Sheridan’s et al. 2025 systematic review findings that trial participation is usually motivated by benefit, altruism or trust [25]. However, trial comprehension was shown to be compromised when participants tended to focus on the positive benefits of the trial, as some perceived that there were would be no additional risks to receiving the novel treatment [22]. These insights indicate a need for trial staff to check participants’ understanding of treatment side-effects, even when participants receive comprehension written information.

The COVID-19 pandemic had varying effects on participants’ trial experiences. Although most participants who took part after the pandemic began felt that their trial experience not been significantly affected, some participants described delays to treatment, reduced access to diagnostics and staging tests and issues with reduced in-person consultations [5]. Similar cancer trials reported that the pandemic had influenced patients receiving later diagnoses and trial treatments because of delayed presentations to primary care, hospital suspensions of non-emergency surgeries, risk of cross-transmission and patient’s fear of infection [26, 27]. Ensuring that participants are provided with the opportunity to discuss ongoing concerns with healthcare professionals through extended consultations including remote sessions has been reported as a method of supporting patients to continue to engage in trials during uncertain times [28].

Overall, participants described broadly positive experiences of the trial. Despite some difficulties with trial comprehension, patients felt that being provided with appropriate information and support prior to recruitment, with ongoing information about tests and treatments, enabled them to feel informed and allay some concerns about treatments and procedures [29, 30]. The opportunity for trial participation was perceived as enabling a higher level of care, more monitoring or a more precise diagnosis than they may have without the trial [31]. Some participants also reported a high level of personal support from clinical and third sector services, which made a positive impact on their overall trial experience, reflecting previous research findings and the need to ensure that all future patients have access to these services [32, 33].

### Strengths and limitations

The longitudinal data provided in-depth insights into recruitment and patient experience of the trial. Although an equal balance of males and females were represented, this did not reflect the percentage of men that develop oesophageal cancer [34] trial. However, no obvious experiential differences were captured between males or females. Initial feedback from recruiting sites highlighted the barriers to participation, exacerbated by the COVID-19 pandemic and provided opportunities to improve trial recruitment strategies. Recruitment to the qualitative study was hampered by the COVID-19 pandemic, which impacted the financial resources and time available to recruit participants, leading to recruitment ending without being able to achieve planned numbers. This affected the breadth of experiences explored across the different trial arms and trial decliners but were not essential when exploring more general trial experiences. This study was unable to further explore the reasons for patients declining the main trial, as none agreed to be referred to the qualitative study. Also, recruitment may have been biased to those healthy enough to participate or with something significant to discuss, rather than the wider cohort, as participants were self-selecting.

### Recommendations

The low levels of qualitative study recruitment illustrated a need for improved recruitment strategies for future qualitative studies embedded in trials. Recruitment could be improved through highlighting the value of integrated qualitative studies, including allowing a real-time assessment of patient experience, that offer opportunities to identify early improvements to trial design. Likewise, a more integrated approach to qualitative elements during trial set up and recruitment processes. This could be enhanced through an opt-out consent form [35, 36] being presented alongside the introduction of the main study. This method may also provide better opportunities for purposive sampling and potentially increase diversity among participants. Qualitative researchers having direct access to the contact details of those that declined the trial may enhance future recruitment. More realistic funding and time allocation for qualitative studies including recruitment at clinical sites could also reflect the complexities of recruitment throughout the trial process.

## Conclusions

Participants of the SCOPE2 qualitative study were generally positive about their overall trial experience. However, trial comprehension was sometimes limited. Improvements to ensure patients have adequate opportunities to discuss and revisit clinical and trial information could enhance patients’ understanding and experience of future trials and procedures. Recruitment to the qualitative study was slow, further impeded by the COVID-19 pandemic, and requires a more fully integrated approach to qualitative recruitment in future trials.

## Supplementary Information

Supplementary Material 1.

Supplementary Material 2.

Supplementary Material 3.

Supplementary Material 4.

## Data Availability

The dataset(s) supporting the conclusions of this article is(are) included within the article (and its additional file(s)); a coding structure is available on request.
