# Supplementary material for: A qualitative study exploring participants’ experiences of the SCOPE2 trial: chemoradiotherapy dose escalation in oesophageal cancer
Source: Trials. 2025 Feb 26;26:70. doi: 10.1186/s13063-025-08768-z (PMC11863524; doi:10.1186/s13063-025-08768-z)
Supplement: Supplementary file 2 — Supplementary Material 2. [file 13063_2025_8768_MOESM2_ESM.pdf]

Supplement 3

Interview Schedule 2

16.0 Embedded Qualitative Study Design

16.1 Rationale

The qualitative component of the SCOPE 2 trial will explore patient experiences and perceptions of participating in a trial of escalated definitive chemoradiotherapy (dCRT) compared with standard dose, and of the two PET driven drug regimes.

16.2 Embedded qualitative study aims [AS PER PROTOCOL]

1. To assess patient experience and perceptions of each dCRT arm of the trial
2. To compare patient views across the dCRT arms of the trial
3. To consider how participants' views change over time spent on treatment
4. To examine the personal impact of treatment on patients' health and wellbeing
5. To understand patients' reasons for declining the trial

Qualitative interviews flowchart

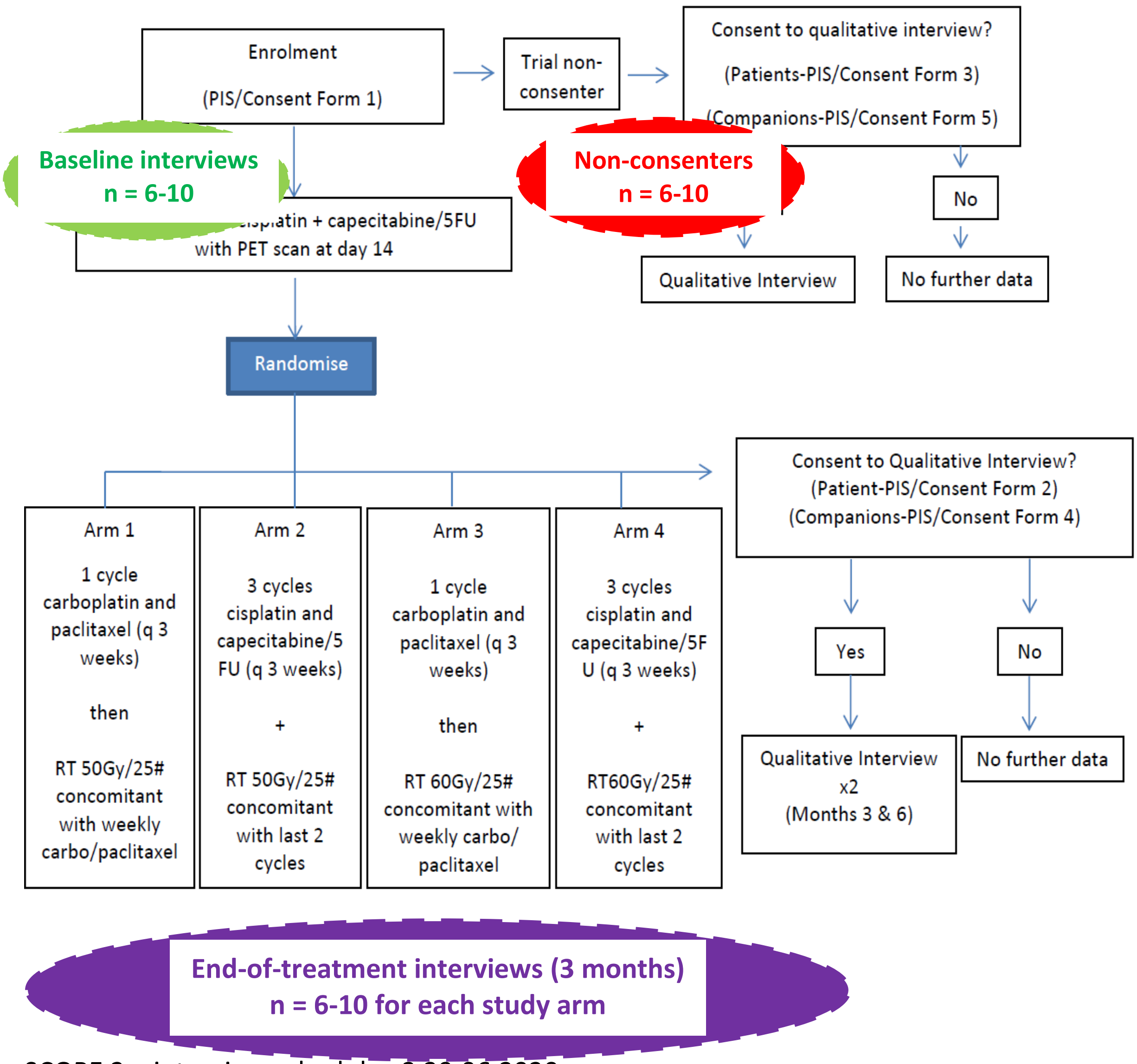

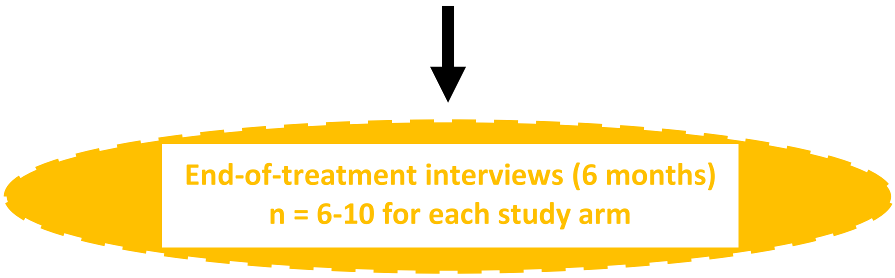

BEFORE THE INTERVIEW

Rapport building

- Thank you for inviting me into your house

Introduction

- Thank you for agreeing to help me with this project – give a few details about the trial (e.g. name, centres, etc)
- I would also like to emphasise that only the study research team will see the information you give me.
- Your name will never be attached to any of them.
- As I mentioned I record the conversation to ease the analysis.
- However, in order for me to do this I need you to have your written consent
- Consenting the companion. As explained your …… can make any point or comment during the interview. However, again for us to be able to use the information given we need to consent ……. as well.
- Last but not least, if anything is not clear, please stop me at any time
- State the purpose of the interview

NON-CONSENTERS INTERVIEW GUIDE

Today we are going to talk about your experience of being invited to take to the SCOPE trial. The interview should take between 30 and 45 minutes. If you need a break at any time please let me know. Are you comfortable to carry on with the interview? (Reassure participants that this is not a test of knowledge – people remember different things)

Specific questions

1. When did you first hear about the trial?
    i. Who explained it to you?

2. What information were given to you?
    i. What did you think the aim of the trial was?
    ii. What was your understanding of the randomisation process?
    iii. Did you discuss the information given with others?
    iv. Did you feel you had enough time to think about the information given?

3. Can you tell me why you preferred not to participate in the trial?
    i. Did you feel that the trial was not what you expected?
    ii. Did you feel supported when making this decision?
    iii. Did you have any concern about turning the trial down?

4. Do you (or someone close to you) have any previous experience of being in a trial?

5. Do you (or someone close to you) have any experience of radiotherapy?
    i. Experience of chemotherapy?

6. Is there anything we could do to make it easier for patients to take part to a similar trial in future?

7. **Concluding questions**
8. I have been asking you many questions, is there anything you would like to ask me?

CONSENTERS BASELINE: INTERVIEW GUIDE

Today we are going to talk about how you have been feeling lately and how you feel about taking part in the trial. The interview should take between 30 and 45 minutes. If you need a break at any time please let me know. Are you comfortable to carry on with the interview? **(Reassure participants that this is not a test of knowledge – people remember different things)**

Specific questions

1. When did you first hear about the trial?
    i. Who explained it to you?

2. What information did you receive?
    i. What did you think the aim of the trial was?
    ii. What was your understanding of the randomisation process?
        a. (Try to explore understanding of equipoise)
        b. (How they feel about uncertainty if raised)
        c. (understanding the different arms of the trial)
    iii. Did you discuss the information given with others?
    iv. Did you feel you had enough time to think about the information given?

3. Can you tell me why you decided to participate in the trial?
    i. What were your main motives for joining?
    ii. Did you feel supported when making this decision?

4. How are you feeling?

Concluding questions

5. I have been asking you many questions, is there anything you would like to ask me?

FOLLOW UP ARMS 1 TO 4 – TIME 1 (3 months): INTERVIEW GUIDE

Rapport building

- Thank you for inviting me again into your house

Introduction

- Thank you for wanting to help me again with the project.
- Similarly to last time I record the conversation to ease the analysis.
- However, I need you to give your written consent
- Consenting the companion. as explained your …… can make any point or comment during the interview. However, again for us to be able to use the information given we need to consent ……. as well.
- Last but not least, if anything is not clear, please stop me at any time

Today we are going to talk about how you have been feeling lately, your experience about the treatment you received and how you feel about taking part in the trial. The interview should take between 30 and 45 minutes. If you need a break at any time please let me know. Are you comfortable to carry on with the interview? (Reassure participants that this is not a test of knowledge – people remember different things)

Specific questions

Joining the trial

1. How have you found being part of a clinical trial so far?
    i. Is what you expected?
    ii. Anything particularly positive?
    iii. Anything negative or that could be improved?
2. Have you had any questions or concerns since you have been on the trial?
    i. Did you speak to somebody about these concerns?

Treatment allocation

3. Have you had all your treatment?
4. What treatment were you on?
5. Have you been given information on how the treatment is working for you on the current state of your illness?
    i. What was your reaction to this information?
    ii. What did you think of the way the information was given to you?
6. How long have you been on your treatment?
7. Is this the treatment that you preferred?
    i. With hindsight, would you have preferred a different treatment?

ii. How did you feel after your PET scan (*avoid this question if the patient was ineligible for pet scan – check with the RN*) (logistics, side effects)

Treatment experiences

8. How have you been feeling
    i. Symptoms
    ii. Psychologically
    iii. Coping (If there are psychological difficulties or coping difficulties, how have they been addressed? Has the participant talked to anyone? Who supports them?)
9. Are you experiencing any symptoms at the moment? How do you manage them (medications, complementary therapies)?
10. Have you been getting any side effects from the treatment you received?

Impact of treatment on quality of life

11. How has your treatment affected your daily life?
    i. What has it stopped you doing? (try to tease out aspects around the logistics of the treatment [e.g. time away from home] and side effects from drugs [side effects/fatigue])
    ii. How have these symptoms affected you family and social life?
12. How is your quality of life since starting the treatment?
13. Does your treatment affect your family/social life?
    i. Time away from home
    ii. Side effects/fatigue
    iii. Withdrawal

Impact of Coronavirus

14. Do you feel that the coronavirus has had any impact on your treatment or recovery?

i. How has the coronavirus affected your quality of life?

ii. Has the coronavirus had an impact on your care or support?

Accessing other services

15. Have been accessing other services? (eg Macmillan or Marie Curie)
    i. Is there any other kinds of support you feel would benefit you?
    ii. Would you know how to access it?

Concluding questions

16. I have been asking you many questions, is there anything you would like to ask me?

FOLLOW UP ARMS 1 TO 4 – TIME 2 (6 months): INTERVIEW GUIDE

Rapport building

- Thank you for inviting me again into your house

Introduction

- Thank you for wanting to help me again with the project.
- Similarly to last time I record the conversation to ease the analysis.
- However, I need you to give your written consent
- Consenting the companion. as explained your …… can make any point or comment during the interview. However, again for us to be able to use the information given we need to consent ……. as well.
- Last but not least, if anything is not clear, please stop me at any time

Today we are going to talk about how you have been feeling lately, how symptoms from treatment might have changed and how you feel about taking part in the trial. The interview should take between 30 and 45 minutes. If you need a break at any time please let me know. Are you comfortable to carry on with the interview? (Reassure participants that this is not a test of knowledge – people remember different things)

Specific questions

The trial follow up

1. What is your understanding of how long you will be in the trial and what happens next?
   i. Follow up at 9, 12, 16, 20, 24 months
   ii. 3, 4, 5 years after you first joined the trial
2. How do you feel about the health care support you have been getting since you joined the trail?
   i. Is this what you expected?

Post-treatment experiences

3. What treatment were you on?
4. How long were you on your treatment?
5. How have you been feeling
   i. Symptoms
   ii. Psychologically
   iii. Coping (If there are psychological difficulties or coping difficulties, how have they been addressed? Has the participant talked to anyone? Who supports them?)
6. Are you experiencing any symptoms at the moment? How do you manage them (medications, complementary therapies)?
7. Have you been getting any side effects from the treatment you received?

8. How did you feel about having a second PET scan? (*avoid this question if the patient was ineligible for pet scan – check with the RN*) (logistics, side effects)

Impact of treatment on quality of life

9. How has your treatment affected your daily life?
   i. What has it stopped you doing? (try to tease out aspects around the logistics of the treatment [e.g. time away from home] and side effects from drugs [side effects/fatigue])
   ii. How have these symptoms affected you family and social life?
10. How is your quality of life since starting the treatment?
11. Does your treatment affect your family/social life?
   i. Time away from home
   ii. Side effects/fatigue
   iii. Withdrawal
12. How would you say that any symptoms and side effects you've experienced changed over the course of your illness?
13. Do you feel better or worse now than you did at the time of the diagnosis
   i. Physically
   ii. Mentally
14. How have you learned to manage your illness?

Impact of coronavirus

15. Do you feel that the coronavirus has had any impact on your treatment or recovery?

i.How has the coronavirus affected your quality of life?

ii. Has the virus had an impact on your care or support?

Accessing other services

16. Have you been accessing other services? (eg Macmillan or Marie Curie)
   i. Is there any other kinds of support you feel would benefit you?
   ii. Would you know how to access it?

Concluding questions

17. I have been asking you many questions, is there anything you would like to ask me?
