# Supplementary material for: A qualitative study exploring participants’ experiences of the SCOPE2 trial: chemoradiotherapy dose escalation in oesophageal cancer
Source: Trials. 2025 Feb 26;26:70. doi: 10.1186/s13063-025-08768-z (PMC11863524; doi:10.1186/s13063-025-08768-z)
Supplement: Supplementary file 3 — Supplementary Material 3. [file 13063_2025_8768_MOESM3_ESM.docx]

| **Findings** | **Illustrative Quotations** |
| --- | --- |
| **Recruitment to the trial** | |
| Motivations to join the trial | |
| Participants often described altruistic reasons for participating in the trial, several were motivated by the possibility of contributing to research, which could improve treatments and services for future generations. Gratitude and a sense of moral duty towards the NHS were also expressed as key motivations for joining the trial. | *I really do think trials are an absolute necessity.* **Participant 6 (Baseline)**  *I was pleased to be asked. Delighted to help national health, I had so much wonderful treatments… if I can give some help back then I am absolutely very pleased to do that.* **Participant 3 (2-3 months)**  *If it helps other people, it will go a long way. Because, I am getting a lot of help, so it will go a long way.* **Participant 10 (Baseline)** |
| Some participants felt that potentially receiving better care and monitoring throughout the trial also encouraged their participation. | *I think we were given indication that there might be an element of better, fuller care.* **Participant 1 (2-3 months)**  *I am so, so, so grateful, I looked through the trial and we are going to be monitored. I don’t think that as a burden. If somebody is going to be looking over my shoulder, if somebody is going to be checking me, if somebody is phoning me up in 3 months time saying “how you feeling”?* **Participant4 (Baseline)**  *I was okay with it because I don’t think there was any extra things to worry about than if I’d have been having normal treatment. Probably was looked after a bit more on the trial.* **Participant5 (3 months)**  *I only knew about my mum’s particular kind of throat cancer and of course, we’ve researched that… but mine’s a different kind of cancer than hers… but we've always followed cancer research since then because we've been interested in it.* **Participant 8 (3 months)**  *Oh, to help other people. If it helps other people, it will go a long way. Because, I am getting a lot of help, so it will go a long way*. **Participant 10 (Baseline)**  *I really do think trials are an absolute necessity.* **Participant 6 (Baseline)**  I think there’s an automatic desire to want to have a better treatment than the one … that may be on offer. **Participant 6 (Baseline)** |
| Joining the trial at times offered hope to participants who had limited treatment options available. Despite some misgivings about chemotherapy, one patient explained that the trial offered a chance for them to regain their basic quality of life. | *I don’t like the idea of destroying all the cells in my body and starting again, no, did I not, but it needs to be done, otherwise I’ll never eat again.* **Participant 8 (6 months)** |
| **Decision to join the trial** |  |
| The provision of sufficient time to consider the trial and discuss and confirm its details with staff was considered important, as patients felt that it could be difficult to absorb all of the information at once, particularly during the challenging time around diagnosis. Also, information provided by healthcare professionals about being able to stop participating, reassured patients about joining the trial. | *The day I made the decision, I don’t think I was taking anything in, but I knew I was making the right decision. I didn’t even look at the paperwork for about another week because there were so many things I was trying to get into my head. So, when [consultant] did ring me, I had a long discussion with him about it, and then I went back to the paperwork. So, I think … it’s a good idea not to frontload people with too much information.* **Participant 6 (Baseline)**  *The information [they] gave me was very good really. It’s all I can say. I mean, cos I don’t know any different. They did advise me as, you know we’d be looked after and I could stop if I wanted to stop it, but they’ve been very good about getting me through it.* **Participant 5 (3 months)** |
| Involving family members in the decision-making process was considered vital for some patients. However, it was also recognised that despite family discussions and relatives’ preferences, ultimately the decision to join the trial was the patients’ to make. | *I think that having cancer doesn’t just affect one person but affects whole of the family, it was one of the things we decided very, early on, that we discuss everything, we talk about everything, whether it was good news, bad news, whatever the procedure was, I wanted my wife, my children, to be a part of that decision. They are very, very happy for the trial, I mean my daughter is a nurse, so you know she understands the solid implications … we all ought to be guinea pigs sometime or other, you know we don’t advance science without it.* **Participant 4 (Baseline)**    *It’s not just me, it’s my husband as well, because he’s been coming along to the meetings and there was lots of questions my husband wanted to ask as well, so … we were treated as a team, … which we are … so, I wasn’t on my own in making that total decision … and also, it’s great if somebody’s been with you… you can go home and discuss it all again… I discussed it with all of my family, because I obviously thought it was something that they needed to know … that we’d made the decision to go with the trial.* **Participant 6 (Baseline)** |
| **Understanding the trial information** | |
| Information provision particularly at the beginning of the trial was perceived as generally useful and patients reported satisfaction with their understanding of the trial and treatment options. Patients felt reassured by the opportunity to discuss their on-going concerns with their hospital trial contacts. The inclusion of family members in trial and healthcare appointments provided additional support during a difficult and often confusing time and enabled patients to gain a better understanding of what was happening. | *Interviewer: how did you feel about that in terms of describing the different treatment options?*  *Patient: oh, very clear, very happy.* **Participant 3 (2-3 months)**  *The thing that come across is everybody, nobody has gilded anything, nobody had hidden anything, everybody has been honest, not blunt, but honest and I have taken on the journey with them as well. I know from every doctor, from every specialist, from every technician that this is where it is. This could happen, these are the side effects… got to tell you all about this… an awful lot of “how do you feel about this?* **Participant 4 (Baseline)**  *There were a lot of other things the doctor … had told me, that weren’t even related to the trial that my husband… was writing things up … [he said] “yeah, she said that to you … yeah, she said that to you” … I wasn’t taking it in.* **Participant 6 (Baseline)**  *I didn’t know really what to expect but I … think it’s just all been very good … very communicative they’ve been … and I couldn’t have wished for anymore.* **Participant 7 (3 months)**  *The doctor explained it fully and they had a lead nurse that continued, I had forms to fill, things like that and they explained fully what was going to happen.* **Participant 9 (6 months).**  *They are very good at [name of hospital], anything I want to know, they do let me know what is going on.* **Participant 10 (Baseline)**  *Interviewer: Do you remember the information that you had, was it clear to you about the trial?*  *Patient: I think it must have been, but I do have my daughters with me and you know, they understand it more than I do and one of them is a nurse.* **Participant 10 (Baseline)** |
| More timely and thorough information would have been helpful to some participants, who were at times unclear about what would happen before, during and after their treatments. This particularly related to how much time the treatment process would take, as they were not always prepared for the length of time, or amount of organisation and resources that were required, especially in relation to radiotherapy. These concerns were more apparent in later interviews, as participants had time to reflect on any information deficits. | *I am completing the radio this week and the chemo care is on next week… and that’s the end … Precisely what happens then, the natural order is and how long they may take and what dates they might be for check-ups and endoscopies and so on. We are little big vague on that.* **Patient 1 (2-3 months)**  *That’s the one thing that I possibly underestimated is how much of your life the treatment takes because I did sort of think will I could carry on working in between.* **Participant 4 (2-3 months)**  *I didn’t know really what to expect but I … think it’s just all been very good … very communicative they’ve been … and I couldn’t have wished for any more.* **Participant 7 (3 months)**  *I think that was possibly one of the things going into the PET scan [I ] read everything about it, read other people’s experiences about it. Knew what was coming… I think for all of these things that was the less frightening thing..., I knew that you would have to sit in the room on your own and you know the reason why, because you know suddenly you are radioactive…, … within the hospital would say “don’t read, don’t read that. Have a look at Macmillan site.”* **Participant 4 (Baseline)** |
| **Experiences of being on the trial** |  |
| Being informed about what would happen during procedures such as PET and CT scans was considered important, as participants felt that full explanations helped allay their concerns. Lack of preparedness for the length of the procedures such as CT scans, as well as insufficient care regarding comfort of the patients during scans was also reported. | *There wasn’t any great care taken to allow for [my] hunch... It was very uncomfortable, and I still got trouble with this arm consequently… certainly I wasn’t told…[A] Little bit more could be said, about lengths of time and divisions all time.* **Patient 1 (2 months)** |
| **Practicalities of the trial** | |
| The pace at which the treatments proceeded once patients joined the trial was perceived as very efficient by certain patients. The high quality of care and efficiency of the testing service was also described. | *Since I’ve been to Oncology… everything has just been zoom, zoom, zoom… The days I went for all the tests, I felt like a VIP, I was being whisked to the bloods, and then to ECG … there was no waiting for anything, everybody was expecting me … I was very impressed.* **Patient 6 (Baseline)**  *The radiotherapy staff … the people who’ve been administering the chemotherapy down there … the meetings I’ve had with the specialists … have been very, very good.* **Participant 7 (3 months)** |
| **Impact of the COVID-19 pandemic** |  |
| Participants reflected on the varying impacts that the Covid-19 pandemic had on their trial experiences. Some felt that their trial experience had not been negatively impacted, whereas others described how the pandemic had delayed their diagnosis, treatment start dates or access to treatments. | *The only thing I would say with Covid was... I may have gone to the GP before I did, if COVID hadn’t been around... But actual treatment, no, I don’t think Covid’s had any effect on it at all…. you couldn’t get an appointment and still can’t. I still haven’t had a face-to-face appointment with my GP at all… then when I did get an appointment, cos I’ve had a diabetic review with the nurse and told her I’ve got symptoms.* **Participant 5 (3 months)**  *I don’t think it affected the … the trial … my problem was the initial diagnosis … there was a lot of delays there.* **Participant 6 (Baseline)**  *I had Covid, and it was difficult at times in hospital because of the restrictions you see, and you had to be so careful, and I think it caused a bit of delay in the radiation because you had to wait for a while, but other than that it was inconvenient, but it did not cause any problems with the treatment.* **Participant 9 (6 months).** |
| **Reflections on the trial** |  |
| A sense of gratitude was expressed by certain participants when reflecting on their opportunity to participate in the trial. The extra monitoring, personalised support and quality of care that they had received from the NHS and third sector professionals was perceived as a particularly positive aspect of participating. | *I have felt that the benefit of the trial is that little bit of extra monitoring, that little bit of extra care.* **Participant 4 (6 months)**  *The vascular team were very, very good. I didn’t realise it at the time but when the consultant came in and said, “oh I think I need to get my friend to come and have a look at this” and another consultant came in and they were sort of discussing, I didn’t realise the implications at the time. It was only when they sort of explained oh you very, very lucky. Had your artery not been elastic enough, because I think, I think they managed to get in one cut in the groin and they were able to get 90% of the thing out and they were able to get the cast of the artery as well.* **Participant 4 (6 months)**  *The dietician’s been out today for an update. They visit rather than just telephone… She’ll ring in a couple of weeks... The advice is ongoing … you know I can ring her if I have any problems. She’s been very helpful.* **Participant 5 (3 months)**  *I’ve found it very good. I found that the people, the staff have been excellent… As soon as I was diagnosed by the local … GP, which was in January, I was put straight in touch with a consultant… and from there… they matched my … medical appointments, they have been absolutely tremendous and … I can’t fault the National Health Service for … at all … and they’ve been absolutely … really brilliant.* **Participant 7 (3 months)**  *The medical staff have really been great, and ... I’ve got all the information … [all] I need to do is pick the phone up and I know I can speak to somebody with any questions … I have been in contact***. Patient 7 (3 months)** |
| A sense of gratitude was expressed by patients when reflecting on their opportunity to participate in the trial. Most patients felt that they were aware of how to access support from trial staff, third sector organisations and the NHS more broadly. | *I am aware of… what’s out there that can help you… I am hoping I am not going to need them for a while.* **Participant1 (3-6 months)**  *If I needed to contact the hospital for any reason, anything that I was concerned about… I had really bad pain here and nothing seemed to stop it… so I rang the hospital and said “what I can take”? I think it was before I started having the morphine patches and they said “can you come down”? It was on Sunday… you wouldn’t get that if I was just going to a normal hospital. You know they are so kind and so concerned down there.. There is no problem at all if you have a concern you just need to ring and they will deal with it.* **Participant2 (3-6 months)**  *I’ve got a nurse… I’ve got her telephone number if I need to ring her, but because I’ve been seeing them every week... And, they have rang me. My bloods have not been as good as they should have been for extra medication and things. So, yeah, it’s been spot on really. I’ve not had anything to worry about. And, I’ve been seeing the doctor twice a week when I was on the radiotherapy; you know just checking everything was alright. So, yeah, I’ve had no concerns at all.* **Participant5 (3 months)**  *I have phone calls from the clinical nurse… sometimes just to ask how I am, he’s helped to make appointments for me, when I’ve had problems making them myself and he’s managed to make everything seamless from one thing to another, which I greatly appreciated, because I was a bit all over the place, especially at the beginning of diagnosis… even the radiotherapy team as well, they’ve been brilliant through it all I’ve felt very supported … if [clinical nurse] thought that maybe I wasn’t getting something, that the Oncologist was saying to me… he always made sure that I left that room, understanding everything… I was given lots of phone numbers, but he’s always been my first port of call, because he just sorts everybody else out for me. So, it’s saved a lot of waiting on hold or waiting for calls back, because he just tries to sort it all out in the background.* **Participant6 (3 months)**  *The medical staff have really been great, and ... I’ve got all the information … I need to do is pick the phone up and I know I can speak to somebody with any questions … I have been in contact.* **Participant7 (3 months)**  *Well, anything that I did have questions or concerns… the head of my research team, personal team… always sorted everything out for me… she made it clear that I could ring her at any time. I went to [support service] across the way I didn’t access it as fully as I might’ve … if I would’ve needed financial help or help in other departments, I probably would've gone there. Participant8 (3 months)*  *I was quite happy…. I could get to the hospital, I could ring if I wanted anything, so I didn’t require anything else… the number was available 24hours a day if you wanted. I did ring them a couple of times with problems and they were very good, they just solved it.* **Participant 9 (6 months).**  *I have been so impressed by that, the pleasantness and the care, the care is good.* **Participant1 (2-3 months)**  *I’m just glad you lot do this type of thing, because I don’t think maybe I might not have done so well without this.* **Participant6 (3 months)**  *It’s been a good experience. Obviously, having the cancer is not a good experience, but I think the trial’s been excellent. I don’t. I have no regrets joining it at all. Participant7 (6 months)* |
| High quality of care was reported by a number of patients, the extra monitoring and personalised support that they had received from the NHS was perceived as particularly positive aspect of participating. | *The support has been great. I mean there’s nothing I could say negative really… I’m just glad I did get on the trial... I felt I was getting a bit of extra care because I was on the trial. And, that does help psychologically because you think I’m getting a little bit extra here. So, yeah, I’m very happy with it all… I’ve spoken to friends who had cancer, you know going back some of them ten years ago, and their experiences weren’t as positive as mine… be proud that you’re part of this, these trials because you know you’re helping people. Participant6 (6 months)*  *I could never afford it… when it was diagnosed first, my dad said “look if it was going to take a long time, why don’t you go private, we’ll pay for it?”… I said “I couldn’t have any better treatment, if I had gone private”. Participant4 (Baseline)* |
