# Supplementary material for: A qualitative study exploring participants’ experiences of the SCOPE2 trial: chemoradiotherapy dose escalation in oesophageal cancer
Source: Trials. 2025 Feb 26;26:70. doi: 10.1186/s13063-025-08768-z (PMC11863524; doi:10.1186/s13063-025-08768-z)
Supplement: Supplementary file 4 — Supplementary Material 4. [file 13063_2025_8768_MOESM4_ESM.docx]

**Supplement 2**

Consolidated criteria for reporting qualitative studies (COREQ): 32-item checklist

| **No** | **Item** | **Guide questions/description** |
| --- | --- | --- |
| **Domain 1: Research team and reflexivity** |  |  |
| Personal Characteristics |  |  |
| 1. | Interviewer/facilitator | Which author/s conducted the interview or focus group?  Dr Daniella Holland-Hart  Dr Mirella Longo |
| 2. | Credentials | What were the researcher's credentials? *E.g. PhD, MD*  PhD |
| 3. | Occupation | What was their occupation at the time of the study?  Research Associate, Cardiff University |
| 4. | Gender | Was the researcher male or female?  Female |
| 5. | Experience and training | What experience or training did the researcher have?  All researchers hold extensive expertise in doing interviews. They all hold an updated GCP certificate and NVivo training and hold PhD’s. |
| Relationship with participants |  |  |
| 6. | Relationship established | Was a relationship established prior to study commencement?  No relationship but the researchers used their research experience and training to introduce the research study and mitigate the asymmetry of information between the two parties. |
| 7. | Participant knowledge of the interviewer | What did the participants know about the researcher? e*.g. personal goals, reasons for doing the research*  Reasons for doing the research |
| 8. | Interviewer characteristics | What characteristics were reported about the interviewer/facilitator? e.g. *Bias, assumptions, reasons and interests in the research topic*  Research expertise |
| **Domain 2: study design** |  |  |
| Theoretical framework |  |  |
| 9. | Methodological orientation and Theory | What methodological orientation was stated to underpin the study? *e.g. grounded theory, discourse analysis, ethnography, phenomenology, content analysis*  Thematic analysis, the conceptual thematic framework used in the study is described in the methodology section. |
| Participant selection |  |  |
| 10. | Sampling | How were participants selected? *e.g. purposive, convenience, consecutive, snowball*  The sample were self-selecting |
| 11. | Method of approach | How were participants approached? e*.g. face-to-face, telephone, mail, email*  Face to face through the research nurse. Then via phone from the researchers. |
| 12. | Sample size | How many participants were in the study?  10 |
| 13. | Non-participation | How many people refused to participate or dropped out? Reasons?  One patient was too unwell to participate in interviews after consenting. |
| Setting |  |  |
| 14. | Setting of data collection | Where was the data collected? e*.g. home, clinic, workplace*  At home or via telephone. |
| 15. | Presence of non-participants | Was anyone else present besides the participants and researchers?  Companions were present in some interviews. |
| 16. | Description of sample | What are the important characteristics of the sample? *e.g. demographic data, date*  Gender, age. |
| Data collection |  |  |
| 17. | Interview guide | Were questions, prompts, guides provided by the authors? Was it pilot tested?  Interview schedules included prompts, which were tested by a senior qualitative researcher (AN). |
| 18. | Repeat interviews | Were repeat interviews carried out? If yes, how many?  No repeat interviews were carried out but follow up interviews were carried out. |
| 19. | Audio/visual recording | Did the research use audio or visual recording to collect the data?  All interviews were audio recorded. |
| 20. | Field notes | Were field notes made during and/or after the interview or focus group?  Field notes were not made during the interviews. |
| 21. | Duration | What was the duration of the interviews or focus group?  Mean average 44 minutes |
| 22. | Data saturation | Was data saturation discussed?  Yes, this was discussed within the team. However, we were unable to reach saturation due to the limited number of interviews. |
| 23. | Transcripts returned | Were transcripts returned to participants for comment and/or correction?  No this was not done. |
| **Domain 3: analysis and findings** |  |  |
| Data analysis |  |  |
| 24. | Number of data coders | How many data coders coded the data?  2 data coders (DHH) and (ML) |
| 25. | Description of the coding tree | Did authors provide a description of the coding tree?  A coding tree is available but not described in the paper. However, the main themes and sub-themes are outlined in Table 4. |
| 26. | Derivation of themes | Were themes identified in advance or derived from the data?  Derived from the data itself |
| 27. | Software | What software, if applicable, was used to manage the data?  NVivo 12 |
| 28. | Participant checking | Did participants provide feedback on the findings?  The patients did not comment but were offered a summary of findings. |
| Reporting |  |  |
| 29. | Quotations presented | Were participant quotations presented to illustrate the themes / findings? Was each quotation identified? e*.g. participant number*  All main themes were illustrated by quotes. The patients are identified by a number and at what stage the interview took place. |
| 30. | Data and findings consistent | Was there consistency between the data presented and the findings?  All main themes were illustrated by quotes, supplementary materials provide further evidence of these points and consistency. |
| 31. | Clarity of major themes | Were major themes clearly presented in the findings?  Major themes formed the basis of the presentation of the qualitative analysis, reflecting the purpose of the overall study (i.e. patient experience of the trial and treatments) and derived from the data itself. |
| 32. | Clarity of minor themes | Is there a description of diverse cases or discussion of minor themes?  Sub themes are also discussed, and examples of divergence between participants are outlined in the main text and additional quotations. |
